# Fracture of the Lower End of the Fibula

**Published:** 1846-08

**Authors:** 


*Fracture of the Lower End of the Fibula.*—M. Robert recommends the following means of distinguishing this accident from a *sprain of the ankle-joint*, after waiting two or three days for the swelling to subside, if necessary. Apply one thumb upon the external malleolus, and the other over the supposed fracture, and transform the fibula into a lever, having its fulcrum at the inferior perineotibial articulation. A certain degree of pressure is to be exerted by the thumb on the malleolus externus. If the fibula is uninjured, its entire length is felt to slightly and uniformly bend under the pressure; but if there be a fracture, the lower fragment moves more or less, and projects under the finger, so that even its form may be distinguished. The fracture in these cases is almost always oblique from above downwards and from behind forwards, occurring at only a short distance from the ankle-joint.

M. R. observes that the fracture may thus always easily be detected, and is surprised the mode has not occurred to others. Dupuytren was not aware of it, but used to seize the leg with one hand and the foot with the other, which means will however only suffice to detect a fracture when situated at a considerable distance from the malleolus.—*Ibid, from Gazette des Hôpitaux.*

